# Indoor Air Quality and Potential Health Risk Impacts of Exposure to Antibiotic Resistant Bacteria in an Office Rooms in Southern Poland

**DOI:** 10.3390/ijerph15112604

**Published:** 2018-11-21

**Authors:** Ewa Brągoszewska, Izabela Biedroń

**Affiliations:** 1 Faculty of Power and Environmental Engineering, Department of Air Protection, Silesian University of Technology, 22B Konarskiego St., 44-100 Gliwice, Poland; 2 Institute for Ecology of Industrial Areas, Environmental Microbiology Unit, 6 Kossutha St., 40-844 Katowice, Poland; izabiedron@gmail.com

**Keywords:** indoor air quality, health risk assessment, bioaerosol, size distribution, antibiotic resistance

## Abstract

The aims of this article are to characterize: the quantity of culturable bacterial aerosol (QCBA) and the quality of culturable bacterial aerosol (QlCBA) in an office building in Southern Poland during the spring. The average concentration of culturable bacterial aerosol (CCBA) in this building ranged from 424 CFU m^−3^ to 821 CFU m^−3^, below Polish proposals for threshold limit values. Size distributions were unimodal, with a peak of particle bacterial aerodynamic diameters less than 3.3 μm, increasing potentially adverse health effects due to their inhalation. The spring office exposure dose (SPED) of bacterial aerosol was estimated. The highest value of SPED was in April (218 CFU kg^−1^), whereas the lowest was in June (113 CFU kg^−1^). Analysis was undertaken to determine the antibiotic resistance of isolated strains and their ability to form biofilms, which may facilitate the spread of antibiotic resistance genes. In the course of the study, it was found that *Staphylococcus xylosus* had the greatest ability to form biofilms, while the strains with the highest antibiotic resistance were *Micrococcus luteus D* and *Macrococcus equipercicus*. Given that mainly antibiotic-sensitive bacteria from bioaerosol were isolated, which transfers resistance genes to their plasmids, this shows the need for increased monitoring of indoor air quality in workplaces.

## 1. Introduction

Indoor air quality (IAQ) has received increasing attention from environmental regulators concerned with improving the comfort, health, and wellbeing of building occupants [1]. For many, the health risks from exposure to indoor air pollution may be greater than those related to outdoor pollution because people spend about 80–90% of their day in indoor environments, of which ~25% is spent at work [2,3,4,5,6]. Actually, almost 30% of office workers complain of health problems, linking them with poor IAQ [7]. Exposure to biological agents in the work environment is associated with a wide range of health effects, including three major groups of diseases: infections, toxic and allergic reactions [8,9,10,11]. Employees in office buildings often share a small space containing a wide spectrum of microorganisms. Human skin, mouths, and nasal cavities contain billions of microorganisms, which can then accumulate in offices. Soil microbes from plants can also be breathed in by office workers or can be transferred to dust particles from the outdoor air [12,13,14].

Many studies about health effects caused by bioaerosol have been conducted in recent years [15,16,17,18]. However, a number of these studies have focused mainly on determining the overall concentration of biological particles in the air and, given the potential of these air pollutants to cause disease, there is insufficient data currently available [19]. Undertaking an effective assessment of exposure to bioaerosols would necessitate data about the size distribution and dose of inhaled air pollutants [19,20,21]. Bioaerosols vary greatly in size from 0.02 to 100 μm and inhaled penetration depth is highly dependent on particle size, wherein the respirable fractions (<3.3 μm) are particularly important in the epidemiology of diseases [22]. The dose of inhaled air pollutants is also a key factor affecting health. Dose refers to the amount of pollutant absorbed or deposited in the body at a given time. Internal dose is the amount of an agent that is absorbed into the body, whereas a biologically effective dose is the amount of pollutant interacting with a target site. However, personal exposure levels and individual susceptibilities are highly variable and this explains why an inhalation dose has not yet been established to characterize the actual risk to workers from exposure to bioaerosols resulting from work activities [23].

Greater understanding of the quality of culturable bacterial aerosol (QlCBA) is also essential to human health. The most commonly occurring groups of bacteria in indoor air are Gram-positive cocci and Gram-positive rods forming endospores (family *Bacillaceae*) [10,16,23,24,25,26,27]. These are of particular importance to public health due to the way their microorganisms are transmitted. In this study, characterization of the isolated strains includes an assessment of biofilm formation, which is an important feature of surface persistence and pathogenesis. Use of antibiotics may accelerate the development of antibiotic resistance genes (ARGs) and bacteria which shade health risks to humans and animals. It is difficult to find an area where ARGs cannot be detected. Considering growing evidence that clinical resistance is intimately associated with environmental ARGs and bacteria, it is quite clear that further research needs to be undertaken to include nonpathogenic or environmental microorganisms [18]. These increase the potential for antibiotic-resistant pollution of bioaerosols. To overcome multi-drug resistance, a greater understanding is required of how bacteria adapt to different habitats, and one form of adaptation to environmental conditions is biofilm. A reservoir of antibiotic resistance results from a complex interplay of factors, including the ecology and physiology of bacteria and the whole set of abiotic conditions. Furthermore, the detection of resistance phenotypes in bacteria which are not a pathogen may offer novel genetic determinants of resistance. Moreover, using molecular methods and detection of a resistance gene is not equivalent with its expression, but it can be evidence of the potential of this gene to spread through the environment. This gene was also tested for resistance to antibiotics, which may help determine the potential health risk to humans if the strain is present in bioaerosols in workplace environments.

The objectives of the present study are to characterize the quantity of culturable bacterial aerosol (QCBA) and the quality of culturable bacterial aerosol (QlCBA) present in office rooms in the Upper Silesia region of Southern Poland during four months of the spring season (March/April/May/June). The research includes three aspects. Firstly, this study aims to investigate the concentration of culturable bacteria aerosol (CCBA) and determine the size distributions of bacterial aerosol. The second aim is to compare the spring office exposure dose (SPED) during the four months of measurement. The third aim is determining the possibility of bioaerosols being a potential source of and reservoir for opportunistic bacteria, and source for resistance gene transmission.

There is a lack of global standards and guidelines for microbiological IAQ. Therefore, this study is a useful tool for developing appropriate control strategies to minimize the adverse effects on health of biological agents in office spaces.

## 2. Materials and Methods

### 2.1. Characteristics of the Sampling Site and Office Building

**Figure 1:**
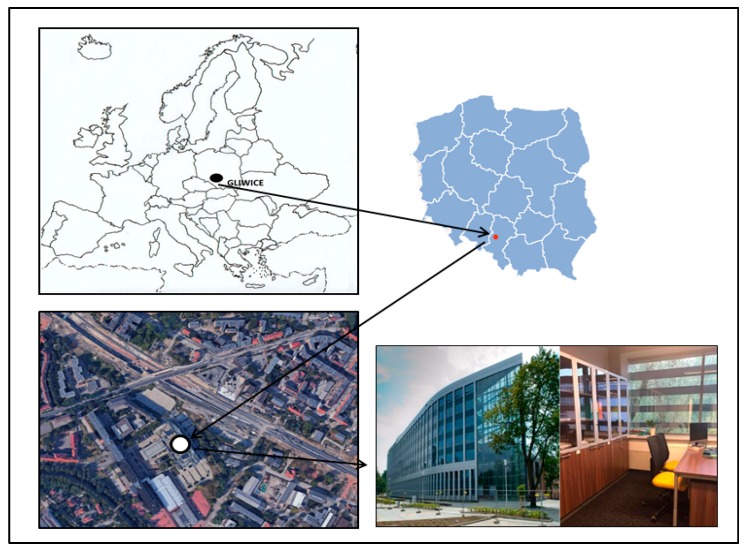
Sampling site location (Map data: 2017© Google, ORION-ME; https://commons.wikimedia.org).

The bioaerosol samples were collected from an office building located in Gliwice (50°17’37.1” N 18°40’54.9” E), Upper Silesia, Southern Poland. The office building was constructed in 2014 and is located in a detached building with seven floors and a usable area of over 14,000 m^2^. The offices are fully carpeted. Cleaning takes place daily before staff arrive. There were one or two occupants per office. Selected offices were naturally ventilated, and the IAQ is primarily maintained by means of stack ventilation and airing through open windows. The exact location of the office building is presented in Figure 1. A detailed description of characteristics of the sampling site and office building can be found in Brągoszewska et al. [23].

### 2.2. Sample Collection

Samples were collected during the spring season (from March to June 2017) using a six-stage Andersen cascade impactor (Thermo Fisher Scientific, Waltham, MA, USA) with cut-off diameters of 7.0, 4.7, 3.3, 2.1, 1.1 and 0.65 μm. The pump ensured a constant flow rate (28.3 L·min^−1^) throughout the impactor. The sampling time was 10 min, following Nevalainen et al. [28,29]. Microorganisms were collected on nutrient media (specific to bacteria) in Petri dishes located at all impactor stages. Trypticase soy agar (TSA) was used, with cycloheximide added to inhibit fungal growth.

We analyzed 3600 Petri dishes with biological material per 4 months (25 days per month, without Sundays and public holidays) in three selected offices. The sampling was collect every of 25 days with 2 repetitions for a day, after 5–6 h of work. Samples of bioaerosol were collected in the centre of each office room at a height of about 1.5 m to simulate aspiration from the human breathing zone. During the measurements, environmental parameters were also recorded (Table 1). The spring season was selected for this research because recent studies of microbiological air pollution conducted in Upper Silesia, Poland, indicate that the highest average concentration of bacteria aerosol is consistently found in this season [8,24,30,31].

### 2.3. Bacterial Strain Selection

Collected airborne bacteria were cultivated at 37 °C for 24 h and total colony forming units were counted. Bacterial strains obtained from each intake were initially identified by observing macroscopic features, such as the appearance of colonies, pigmentation, etc., and microscopic characteristics (bacterial morphology, motility, and reaction to Gram staining, etc.). Further steps focused on selecting a group of microorganisms present in each of the tested samples. For this purpose, a comparative analysis of the created feature matrix was used. A series of screening analyses led to the acquisition of strains of bacteria, present during each collection. The selected microorganisms were then identified, and their antibiotic resistance and biofilm formation were determined.

### 2.4. Identification of Selected Bacteria

Selected strains were identified on a Biolog GEN III (Biolog, Hayward, CA, USA) microtiter plate, containing 71 different carbon sources and 23 chemical sensitivity assays. After 24-h incubation on TSA plates, single colonies were transferred into inoculating fluid. The bacterial suspension was adjusted to 95% transmittance using a Biolog turbidimeter. Then, 150 μL of the suspension was dispensed into each well of the Biolog GEN III microplate. Inoculated plates were monitored via Biolog’s OmniLog software for 48 h at 37 °C with measurements taken every 15 min. Their identity was confirmed using the Biolog GEN III MicroPlate, based on phenotypic similarity to the control strains in the BIOLOG database.

#### Biofilm Formation Assay

Biofilms were formed in 96-well microtiter plates as described by Srednik et al. [32]. The colonies were suspended in fresh Luria–Bertani (LB) broth to 0.5 McFarland standards, and 200 μL was deposited on the microtiter plates in triplicate. After incubation for 24 h at 37 °C, the microtiter plates were washed three times with phosphate-buffered saline (PBS), and the liquids were removed by aspiration and air-dried. The bacterial biofilm biomass was stained with 0.1% safranin and, after 10 min, was washed with distilled water and air-dried. A solution of ethanol and glacial acetic acid (volume ratio of 1:1) was added. The concentration of the released strain was determined by measuring the optical density of the solution at 490 nm using a microtiter plate reader. The sterile LB broth was used as the control. The experiment was performed in triplicate and average values were obtained. The ability of the isolate to form a biofilm was classified by the scales of biofilm formation given by Zhang et al. [33]. Where criteria for the biofilm capacity were specified, this was based on the OD value of the control (Ac). In this study, the average Ac value was 0.11 at 490 nm. The scale of the isolate to form a biofilm was as follows: negative (−) A ≤ Ac; weakly positive (+) Ac < A ≤ 2Ac; moderately positive (++), 2Ac < A ≤ 4Ac; and strongly positive (+++), A > 4Ac.

### 2.5. Antimicrobial Susceptibility Tests and the Detection of Antimicrobial Resistance Genes

#### Antibiotic Susceptibility Test

Disc diffusion method for antimicrobial susceptibility testing was carried out according to the Kirby-Bauer Disk Diffusion Susceptibility Test Protocol [34]. The inoculated Petri dishes were incubated at 30 °C for 24 h. After incubation, areas of inhibited growth were measured according to a three-stage scale in order to assess bacterial resistance to antibiotics. If the diameter of growth inhibition <15 mm, then the bacteria were resistant to antibiotics (R); a growth inhibition diameter of between 16 and 25 mm meant that the bacteria had an intermediate level of resistance to antibiotics (I); while a growth inhibition diameter >25 mm meant that the bacteria were sensitive to antibiotics (S). Three repetitions of each antibiotics test were performed. The results of the antibiotics insusceptibility test are presented in Table 2. The method was used to select the most antibiotic-resistant isolate within each of the selected species.

### 2.6. Detection of Antimicrobial Resistance Genes

#### 2.6.1. DNA Isolation

A single colony of each strain was inoculated from Mueller–Hinton agar (MHA) into 30 mL of Luria–Bertani (BTL) broth and incubated for 24 h at 37 °C after being shaken. Cells from the overnight culture were harvested by centrifugation at 5000 rpm for five minutes. The supernatant was then decanted. DNA from the isolated colonies was prepared using the Genomic Mini preparation kit (A & A Biotechnology) in accordance with the manufacturer’s instructions.

#### 2.6.2. Multiplex PCR for the Detection of Selected Antibiotic Resistance Genes

The multiplex PCR reaction was performed in a solution 50 μL in volume using the PCR TaqNova-RED mix, which contained the following: 1XPCR TaqNova-RED mix (Blirt); 200 nM concentrations of the primers *ermA*, *aacA-aphD*, *tekK*, *vatB*, and *mecA*, as well as approximately 5 ng of template DNA. The PCR amplifications were performed in a Mastercycler Nexus Gradient GSX1 thermocycler (Eppendorf) with the following parameters: predenaturation for two minutes at 94 °C; 30 cycles of 94 °C for 30 s; 55 °C for 30 s and 72 °C for 30 s; post extension for four minutes at 72 °C; and soaking at 4 °C. The PCR reaction mixtures (10 μL) were electrophoresed (Casting System Compact Biometra) on 2% agarose gel at 100 V and visualized with 0.5 μg/mL ethidium bromide. The PCR primers used to detect five antibiotic-resistant genes in a multiplex PCR reaction are listed in Table 3.

### 2.7. Statistical Analysis

All statistical analyses were performed using the statistical package Statistica 12 (StatSoft). The concentration values were presented as the mean values and standard deviation. Given that the data were not normally distributed (analysed with the Shapiro–Wilk test), a nonparametric method was employed. The Mann–Whitney U test was applied to assess monthly differences of microorganism concentrations at the sampling site. A statistical significance level of *α* = 0.05 (*p* < 0.05) was used throughout the study.

## 3. Results and Discussion

### 3.1. The Quantity of Culturable Bacterial Aerosol (QCBA)

#### 3.1.1. Concentration of Culturable Bacterial Aerosol (CCBA)

Table 4 presents the average concentrations of culturable bacterial aerosol collected in the indoor office air in Southern Poland during the spring season. The month average levels of CCBA in the office rooms ranged from approximately 424 to 821 CFU m^−3^. The results of the Mann–Whitney U test showed that there was a significant difference between months (*p* < 0.05) such that only the difference between levels of bacterial aerosols collected in May versus June were statistically non-significant (*p* > 0.05).

The concentrations of airborne bacteria recorded in the examined office buildings in Gliwice were similar to those published in other reports. For example, the average concentration of bacteria in the office building in central Poland was 600 CFU m^−3^ [35], as compared to an office building in Hong Kong which has been measured at 580 CFU m^−3^ [36]. A significantly lower concentration level of bacterial aerosol has been found in an office building in Warsaw during the winter season, where the CCBA was 84 CFU m^−3^ [7].

Exposure to microorganisms suspended in the air is associated with a wide range of major, adverse health effects. However, there is no international standard available for maximum levels of bioaerosols in the air, due to variations in human responses to exposure and difficulties in recovering potentially hazardous microorganisms in routine sampling [37]. Work conducted by a WHO expert group on assessment of the health risks of biological agents in indoor environments has suggested that the total microbial load should not exceed 1000 CFU m^−3^ [38], whereas Polish proposals for the mesophilic bacteria suggest 5000 CFU m^−3^ for public service buildings [27]. The bacterial load obtained in this study is below proposed standards.

#### 3.1.2. Size Distribution of Bacterial Aerosol

**Figure 2:**
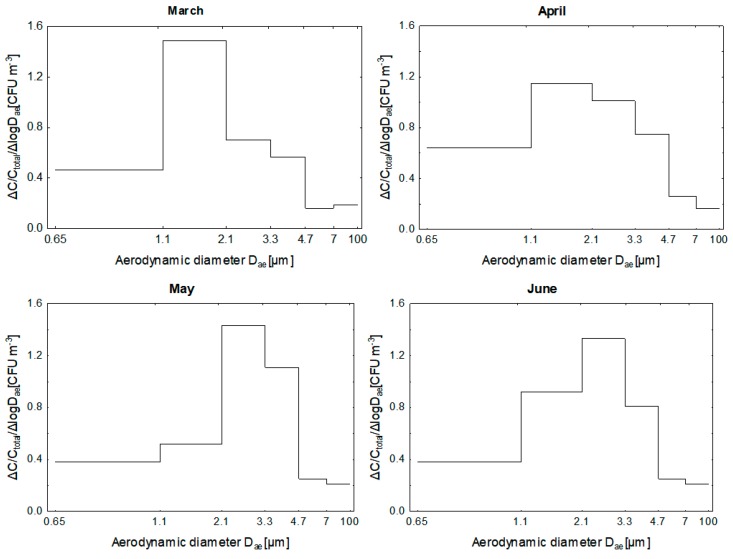
Size distribution of the bacterial aerosol in the offices indoor air. D_ae_—aerodynamic diameter; ΔC—concentration of bacterial aerosol on particular stage of 6-stage Andersen impactor; C_total_—total concentration of bacterial aerosol; Δ log D_ae_—logarithm of differences of cut-off diameters for particular stage of 6-stage Andersen Impactor.

The size distributions of airborne bacteria obtained in four months (March/April/May/June) reached maximum concentration in the air at the range of diameters <3.3 μm (Figure 2).

Size distributions obtained in all four months in spring season were unimodal, with a peak falling in the range of particle bacterial aerodynamic diameters in the range of 1.1 to 2.1 μm in the March/April, and of 2.1 to 3.3 μm in May/June. These respirable particles are mostly deposit either in the tracheal or alveolar region of the lungs.

The examination of the monthly distributions of bacterial size aerosol indicates that, during cooler months, the particle size distribution characterized by an increase in the share of fine and a decrease in the share of coarse fractions. This shape of size distribution may indicate that the particles of airborne bacteria during March and April are relatively fresh, and mostly human origin. In addition during these months due to the low temperature outside offices were not practically ventilated by opening windows. In turn, in the warmer months (May/June) there are very good conditions for the growth of bacterial particles primarily due to the formation of large agglomerates of cells as confirmed by our previous research [8].

#### 3.1.3. Spring Office Exposure Dose (SPED).

Bacterial aerosol exposure dose (SPED) has been calculated on the basis of the EPA’s Exposure Factors Handbook [39]. We used in the calculations of the inhaled dose of airborne bacteria average concentration of bacterial aerosol in every month of spring season. The calculations were based on the following equation:

(1) $$\mathrm{SPED}=\frac{C\cdot \mathrm{IR}\cdot \mathrm{IEF}}{\mathrm{BW}}$$

where:

SPED—is bacterial aerosol exposure dose in office, CFU kg^−1^
*C*—is bacterial aerosol concentration, CFU m^−^³
*IEF*—is indoor exposure fraction: hours spent a day in office, concerning diverse activity patterns hour (in sum on average: eight hours)
*IR*—is inhalation rate coefficient characteristic for selected activity levels, m³/day [39]
*BW*—is mean body weight, kg.

A staff office time-budget survey was used to elicit information about staff activities during the day and a summary of the information obtained from the application of this questionnaire can be found in Brągoszewska et al. [23]. The calculated results of the SPED are presented in Table 5.

The highest value of SPED is inhaled by the staff in offices during April and March. One of the reasons may be that these months were the coolest during this research and offices were rarely ventilated. The calculated average dose in spring is comparable to the dose absorbed by adults inside homes in Upper Silesia, Southern Poland (175.4 CFU kg^−1^) [40]. Furthermore, the bacterial dose inhaled by staff in the studied office building during spring months is nearly twice as low as the dose absorbed by the staff of a nursery school in the same season in Southern Poland (272–309 CFU kg^−1^), where 27 people were present in the room during measurements [41].

### 3.2. The Quality of Culturable Bacterial Aerosol (QlCBA)

#### Identification of Bacterial Aerosol

From all air samples examined, a total of 1762 bacterial strains were isolated. Following analysis of collected data, a significant advantage of Gram-positive isolates was found. In office rooms, 86.4% of isolates were gram-positive microorganisms (56.6% cocci and 43.4% rods). Gram-negative strains were also isolated but they were not present in all samples. The isolates presented in each sample cycle were subjected to microbiological analysis: colony morphology, Gram stain, cell morphology. Seven groups of strains present in each harvesting cycle were obtained in the final screening analysis. Representatives with the highest antibiotic resistance in their group were selected for further analysis. The results of identification using the Gen III Biolog Omnilog system are presented in the Table 6.

The bacteria isolated in this research are some of the most commonly found in indoor urban environments (offices, schools, and residential buildings, etc.), which is in accordance with data from the literature [24,25,42,43]. Generally, the air inside the office buildings studied does not constitute a level of bacterial aerosol exposure that will pose an immediate threat of acute health problems; however, prolonged inhalation of these kinds of doses of airborne bacteria may cause some adverse health effects, especially in sensitive individuals [44].

Results indicate that the isolated strains have a diversified pattern of antibiotic resistance (Table 7). The strain showing the highest sensitivity was *Staphylococcus xylosus*, which was resistant to 8.33% of the substances tested. The highest antibiotic resistance was found in *Macrococcus equipercicus* and *Micrococcus luteus D* (55.56%).

**Figure 3:**
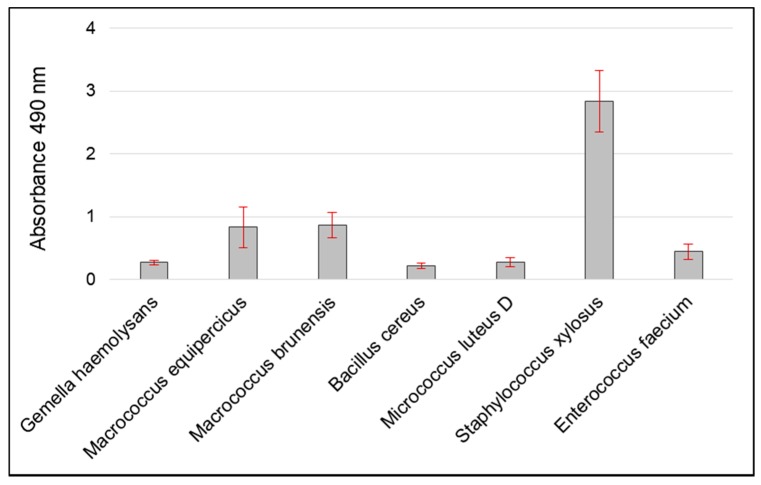
Biofilm formation by the isolated strains. Bars represent the standard deviation.

**Figure 4:**
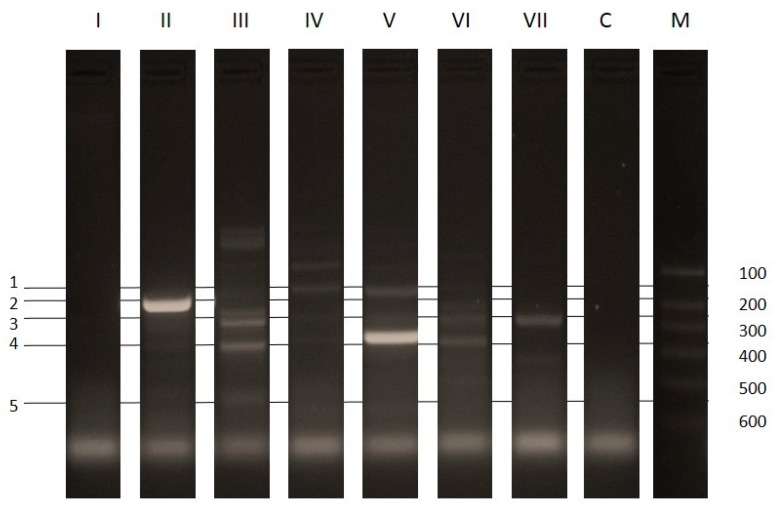
Multiplex PCR of plasmid DNA: Lane I–VII strains (I—*Gemella haemolysans*; II—*Macrococcus equipercicus*; III—*Macrococcus brunensis*; IV—*Bacillus cereus*; V—*Micrococcus luteus D;* VI—*Staphylococcus xylosus*; VII—*Enterococcus faecium*), lane C: negative control - without template DNA; lane M: 100bp DNA ladder. Line 1–5 indicate the size of the amplification products:1-vatB (136bp), 2-ermA (190bp), 3-aacA-aphD (227bp), 4-tekK (360bp), 5-mecA (532bp).

*Gemella haemolysans*, being isolated in an office room is susceptible to ampicillin as repeatedly described in strains isolated in hospital rooms or directly from patients [45]. However, its presence in office rooms can be explained by the fact that it is a commensal of the upper respiratory tract of humans. This bacterium is also isolated from multi-species biofilms which protect against the effects of antibiotics. The research here indicates that it is a strain with low biofilm capacity (OD_490_ = 0.27 ± 0.03) (Figure 3), which means that it can be assumed to live in a biofilm developed as a result of other microorganisms. However, in this study, no difficulties were encountered with cultivation of the strain in a bloodless medium, contrary to the results described by Vandeplassche [46]. In both antibiotic resistance and biofilm analysis, the strain was propagated without any difficulty. *Macrococcus equipercicus* and *Macrococcus brunensis* have a high biofilm capacity (OD_490_ = 0.84 ± 0.32 and OD_490_ = 0.87 ± 0.21) (Figure 3). Resistance to erythromycin in *Macrococcus equipercicus* has been confirmed by both the disk diffusion method and the detection of the gene *ermA* present in the plasmid DNA (Table 7, Figure 4). Gentamicin and ofloxacin resistance in *Macrococcus brunensis* were confirmed by both the disk diffusion method and the detection of the gene *aacA-aphD* and *mecA* present in the plasmid DNA. Despite the sensitivity of *Macrococcus brunensis* to two antibiotics of the genus tetracycline (doxycycline, minocycline), the *tetK* gene was detected. This may be due to the fact that the *tetK* gene expression product induces less resistance to tetracycline derivatives which are doxycycline and minocycline [47]. *Tet* genes are characterized by high mobility, both in Gram positive and Gram negative bacteria [48] and strains with high adhesion ability may affect the spread of this gene among other microorganisms within the biofilm [49].

*B. cereus* was resistant to 14 of the antibiotics tested (Table 7). In isolated plasmid DNA, the *vatB aacA-aphD* and *tekK* genes were identified (Figure 4). In the case of the *aacA-aphD* gene, there was no resistance to gentamicin in the disk diffusion study. These genes are present in the plasmid DNA but may not be amplified, or not expressed or only expressed at a low level. Although the bacteria is described in the literature as biofilm forming [50], this study has found the isolate to be characterized by low adhesion capacity with an OD_490_ value of 0.22 ± 0.04 (Figure 3). The biofilm produced by *B. cereus* can take the form of a pellet floating on the surface of the medium, or in the initial stages create a very weakly bonded structure with the surface [51]. The applied method detects the biofilm produced on the surface of the well, and the strain’s characteristics may be the cause of low biofilm detection in our research.

Results of the disk diffusion method indicate that *Micrococcus luteus D* was resistant to the largest number of antibiotics tested (55.6%) (Table 7). As a result of the analysis of plasmid DNA, the genes *ermA*, *aacA-aphD*, *tekK* were found (Figure 4). However, in this case, there may also be a lack of amplification or low amplification, resulting in sensitivity of the strain to erythromycin, gentamicin, and tetracyclines, respectively. The strain was characterized by a low adhesion capacity (OD_490_ = 0.26 ± 0.07) (Figure 3). This has also been indicated in other research by Górny, where the strain was isolated only from air with no isolates found from surface samples [52].

According to some publications [53,54,55,56,57,58,59], the phenotypic identification of isolated *S. xylosus* strains may be problematic. This is due to the limited number of permanent traits by which a staphylococcus species can be distinguished; another difficulty is also heterogeneity within the *S. xylosus* species [60,61]. Therefore, in addition to standard identification methods, the BIOLOG system was also used for identification purposes in this study. It has been reported that *S. xylosus* may adapt to different environments due to its capability to form biofilms on biotic and abiotic surfaces, such as glass, polystyrene, and steel [62]. What has been confirmed in this research, is a very high OD_490_ value of 2.84 ± 0.48 (Figure 3), indicating a high level of adhesion to the surface. The disk diffusion results show complete resistance of the isolated strain only to aztreonam, metronidazole and nalidixic acid which are examples of the natural resistance present in Gram-positive bacteria (Table 7). However, in their plasmid DNA, a positive result was obtained for the gene *erm A*, which is responsible for the phenotype of resistance to erythromycin, clindamycin, and *tetK* (associated with resistance to tetracycline), as well as *aacA-aphD* (associated with resistance to gentamicin) (Figure 4). Qi et al. [63] note that for some strains, low resistance to antibiotics is compensated by the high biofilm capacity. The research discussed here also observed this with *S. xylosus* results, but only in isolated strains.

*Enterococcus faecium* in office rooms was usually detected on surfaces such as rugs or as a component of dust [64]. Analysis of the antibiotic resistance strain has revealed resistance to a range of antibiotic substances (44.4%) (Table 7), but no Vancomycin-Resistant *Enterococcus faecium* (VRE) was found in office rooms. Among the most common resistance (penicillin, erythromycin, and ampicillin) reported by Dupre et al. [65], the isolated strain was resistant to erythromycin only. Plasmid DNA identified the *aacA-aphD* gene (Figure 4), the presence of which was not associated with resistance of the strain to gentamicin. The strain was characterized by a low adhesion capacity (OD_490_ = 0.44 ± 0.12) (Figure 3).

Although all the isolated strains showed differing resistance to antibiotics, it should be noted that in the natural environment, microorganisms are believed to use antibiotics, which are secondary metabolites, for microbial cell defence, inhibiting the growth of competitors [66]. However, many bacteria have become multidrug-resistant via natural or acquired means. The increasing use of antibiotics may accelerate the development of antibiotic resistance genes and change the equilibrium between fully sensitive and resistant bacteria [67]. Various antibiotic resistance genes, encoding resistance to a broad range of antibiotics, have been found in microorganisms located not only in hospital wastewater but also in sewage, wastewater treatment plants, surface water, groundwater and even drinking water [68]. Indeed, it is difficult to find an area in which antibiotic resistance genes cannot be detected [69]. Resistance can be transferred between bacteria by three genetic mechanisms responsible for HGT (horizontal gene transfer): transformation, transduction, and conjugation, involving cell-to-cell contact through a pilus [70,71]. In a bacterial biofilm, genetic recombination is easily transmitted through HGT. This form of genetic recombination involves the transfer of genetic material from a donor to a recipient, and requires that they both share the same space, but not necessarily the same species. These genetic elements contain information about metabolic capabilities, virulence expression and antibiotic resistance [72]. HGT is widely recognized as the mechanism responsible for the widespread distribution of bacterial antibiotic resistance [73].

Characterization of the isolated strains in this study supports the claim that, although they do not show antibiotic resistance, it carries genes of resistance in its genetic material. This can be particularly dangerous with biofilms, because HGT allows for gene exchange among bacteria, both between those of the same strain and between different species in biofilms. It is an important mechanism that allows bacteria to adapt to changing environmental conditions [69].

Bacteria in a biofilm produce large amounts of extracellular polymer substances (EPS) which influence biofilm integrity, both in terms of physiology and structure, as well as having a direct impact on the physical, chemical and biological properties of the biofilm. The extracellular matrix, besides maintaining a stable microconsortium of different species, is used to protect against harmful environmental conditions, facilitate HGT and enable intercellular communication, which allows for regulation of the gene expression associated with the temporary adaptation to environmental conditions [74,75,76].

Most research currently focuses on the search for microorganisms in the aquatic environment. However, there is also growing interest in the presence of antibiotic resistance genes among bioaerosol microorganisms in work premises [77,78,79]. It is worth noting the potential of microorganisms in office bioaerosols, which, with their concomitant biofilm capacity, may inhabit air filters in air conditioners or other available moist surfaces.

## 4. Conclusions

The study of the quantity of culturable bacterial aerosol (QCBA) and the quality of culturable bacterial aerosol (QlCBA) was carried out in office rooms in Upper Silesia during the spring season (March/April/May/June).

The average concentration of culturable bacterial aerosol (CCBA) in the office building was 653 CFU m^−3^; the highest value of average CCBA was found in April (821 CFU m^−3^) when the office rooms have not been ventilated by opening windows, and the lowest value was observed in June (424 CFU m^−3^) when the offices were well-ventilated. Our study clearly suggests that it is very important to improve the microbiological quality of air in office spaces by providing adequate ventilation.

The size distribution of bacterial aerosol indicates that biological particles less than 3.3 μm contributed up to 80% of the total concentration of bacterial aerosol inside the building being studied, increasing the health risk for exposed personnel in the office building.

The spring office building exposure dose (SPED) of bioaerosols was estimated monthly for the staff of this building. The highest value of SPED was obtained in April for staff working in offices with natural ventilation (218 CFU kg^−1^), while the lowest value of SPED was observed in June (about 113 CFU kg^−1^).

The content and diversity of antibiotic resistance genes in the environment has increased significantly in recent years. It is believed that this has resulted from the widespread use of antibiotics. Nowadays, not only are antibiotics considered to be a form of environmental pollution, but also the genetic coding for antibiotic-resistant bacteria. The occurrence and spread of antibiotic-resistant bacteria has been extensively reported and is considered to be a serious and pressing public health problem worldwide, and not only in a clinical environment. The isolation of mostly antibiotic-sensitive bacteria from bioaerosols, which carry resistance genes on their plasmids, demonstrates the need for better monitoring of air distribution systems and increased awareness of the interactions in biofilms which can form on moist air filter surfaces.

This research confirms that, even in the case of strains not considered to be opportunistic pathogens, these organisms can act as a source of antibiotic resistance in the environment. They may pose a health threat even in such a unique environment as bioaerosols. Therefore, particular attention should be paid to analysing the antibiotic resistance patterns of bacterial strains isolated from different offices.

In the analysis of aquatic environments, antibiotic resistance genes are treated as pollution. This should also be taken into consideration for air quality analyses. Another important aspect is the degree to which these pollutants can be detected; this study has shown that the classic disk diffusion method is only useful to determine the antibiotic resistance of the strain. It in no way provides an answer as to whether the microorganism can be a carrier of antibiotic resistance genes.

Air-control measures are crucial for reducing dissemination of airborne biological particles in workplaces. The research presented here highlights the need to implement best approaches to reducing microbiological air pollution in office spaces. However, further studies are needed to measure and monitor human exposure in workplaces and to relate this to health outcomes.

## Figures and Tables

**Table 1:** Descriptive statistics of indoor air quality (IAQ) environmental conditions in office building by month during sample collection.

Environmental Parameters, Mean ± SD ^1^				
Month	Indoor Temperature, °C	Indoor Relative Humidity (RH), %	Outdoor Temperature, °C	Outdoor Relative Humidity (RH), %
March	19.2 ± 4.1	31.2 ± 2.1	14.1 ± 1.1	76.0 ± 12.7
April	19.8 ± 6.1	34.8 ± 3.2	16.4 ± 2.1	77.8 ± 11.2
May	23.2 ± 6.4	32.4 ± 4.0	19.1 ± 3.2	72.6 ± 12.6
June	24.1 ± 6.2	30.2 ± 2.2	22.2 ± 2.4	71.6 ± 8.1

^1^ SD—Standard deviation.

**Table 2:** Antibiotics used in susceptibility test.

Group of Antibiotics	Antibiotic	Dose (mg)
Aminocoumarin	Novobiocin	30
	Amikacin	30
	Gentamicin	120
	Gentamicin	200
	Neomycin	30
	Netilmicin	30
	Tobramycin	30
Carbapenems	Doripenem	10
	Ertapenem	10
	Imipenem	10
Cephalosporins	Cefaclor	30
	Cefadroxil	30
	Cefepime	30
	Cefoxitin	30
	Ceftaroline	5
	Ceftazidime	30
Drugs against mycobacteria	Rifampicin	30
Glycopeptides	Teicolpanin	30
	Vancomycin	30
Macrolides	Azithromycin	15
	Erythromycin	30
Monobactams	Aztreonam	30
Nitrofurans	Nitrofurantoin	300
others	Metronidazole	50
	Mupirocin	200
	Trimethoprim	5
Penicillins	Amoxycillin	30
	Ampicillin	25
	Piperacillin	100
	Ticarcillin	75
Quinolones/Fluoroquinolones	Ciprofloxacin	10
	Nalidixic acid	30
	Norfloxacin	10
	Ofloxacin	5
Sulfonamides	Trimethoprim/sulph	25
Tetracyclines	Doxycycline	30
	Minocycline	30

**Table 3:** The primers used in the PCR reaction and size of the amplicons.

Target	Primer	Sequence (5’ to 3’)	Amplicon Size (bp)
*aacA-aphD*	aacA-aphD1 aacA-aphD2	TAA TCC AAG AGC AAT AAG GGC GCC ACA CTA TCA TAA CCA CTA	227
*ermA*	ermA1 ermA2	AAG CGG TAA ACC CCT CTG A TTC GCA AAT CCC TTC TCA AC	190
*tekK*	tekK1 tekK2	GTA GCG ACA ATA GGT AAT AGT GTA GTG ACA ATA AAC CTC CTA	360
*mecA*	mecA1 mecA2	AAA ATC GAT GGT AAA GGT TGG C AGT TCT GCA GTA CCG GAT TTG C	532
*vatB*	varB1 vatB2	GCT GCG AAT TCA GTT GTT ACA CTG ACC AAT CCC ACC ATT TTA	136

**Table 4:** Average concentrations of culturable bacterial aerosol (CCBA) in offices (CFU m^−3^).

Month	CCBA	SD ^1^	CCBA _max_	CCBA _min_
March	786	118.14	852	289
April	821	141.21	946	324
May	582	117.47	774	126
June	424	96.24	581	114
Average spring CCBA	653.25	118.26	788.25	213.25

^1^ SD—Standard deviation.

**Table 5:** Calculated exposure dose spring office exposure dose (SPED) of bacterial aerosol inhaled by staff of office building in four months of spring season.

Month	SPED—Office Building Exposure Dose (CFU kg^−1^)
March	210
April	218
May	155
June	113
Average SPED	174

**Table 6:** Species of bacteria isolated from indoor office air.

Species of Isolated Bacteria
*Macrococcus equipercicus*
*Macrococcus brunensis*
*Micrococcus luteus D*
*Staphylococcus xylosus*
*Gemella haemolysans*
*Enterococcus faecium*
*Bacillus cereus*

**Table 7:** Antibiotic resistance pattern on disk diffusion method.

Bacteria	Antibiotics Resistance
*Gemella haemolysans*	Vancomycin, Azithromycin, Erythromycin, Aztreonam, Nitrofurantoin, Metronidazole, Mupirocin, Ticarcillin, Nalidixic acid, Norfloxacin
*Macrococcus equipercicus*	Neomycin, Tobramycin, Ertapenem, Imipenem, Cefaclor, Cefadroxil, Cefepime, Cefoxitin, Ceftazidime, Teicolpanin, Azithromycin, Erythromycin, Aztreonam, Metronidazole, Trimethoprim, Amoxycillin, Ampicillin, Piperacillin, Ticarcillin, Nalidixic acid
*Macrococcus brunensis*	Amikacin, Gentamicin, Doripenem, Imipenem, Cefaclor, Cefepime, Ceftaroline, Rifampicin, Azithromycin, Erythromycin, Aztreonam, Metronidazole, Amoxycillin, Ampicillin, Ofloxacin
*Bacillus cereus*	Doripenem, Ertapenem, Cefaclor, Cefadroxil, Cefepime, Cefoxitin, Ceftaroline, Ceftazidime, Erythromycin, Aztreonam, Metronidazole, Ciprofloxacin, Nalidixic acid, Doxycycline
*Micrococcus luteus D*	Doripenem, Ertapenem, Cefaclor, Cefadroxil, Cefepime, Cefoxitin, Ceftaroline, Ceftazidime, Teicolpanin, Nitrofurantoin, Metronidazole, Mupirocin, Trimethoprim, Amoxycillin, Ampicillin, Piperacillin, Ticarcillin, Norfloxacin, Trimethoprim/sulph
*Staphylococcus xylosus*	Aztreonam, Metronidazole, Nalidixic acid
*Enterococcus faecium*	Amikacin, Neomycin, Netilmicin, Tobramycin, Ertapenem, Cefadroxil, Cefepime, Cefoxitin, Ceftazidime, Azithromycin, Erythromycin, Aztreonam, Metronidazole
